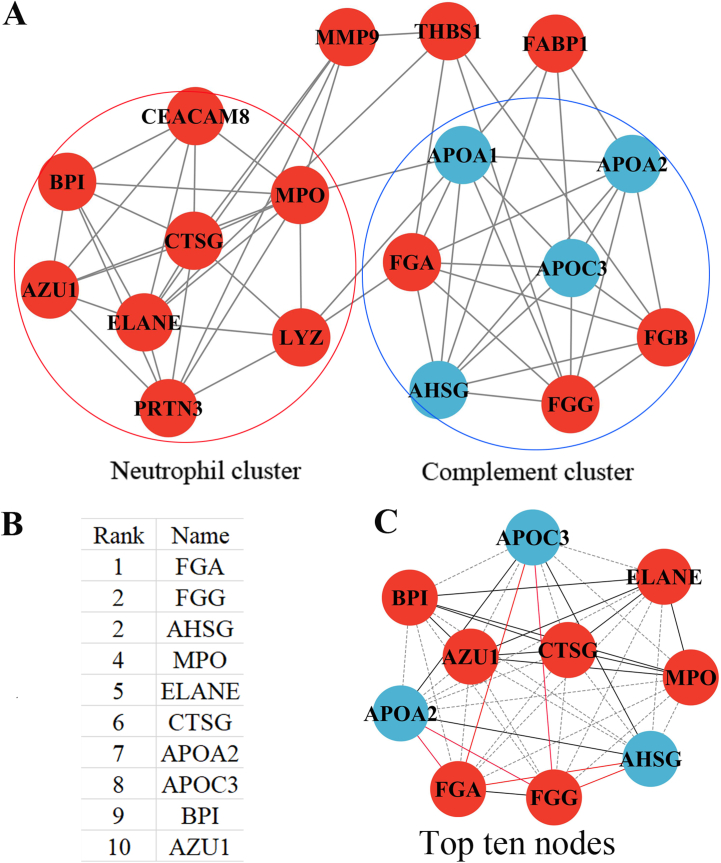# Proteomic lung analysis revealed hyper-activation of neutrophil extracellular trap formation in cases of fatal COVID-19

**DOI:** 10.1016/j.heliyon.2024.e31878

**Published:** 2024-05-24

**Authors:** Shu Song, Liyan Zeng, Jingjing Xu, Lei Shi, Lingqing Lu, Yun Ling, Lijun Zhang

**Affiliations:** a Shanghai Public Health Clinical Center, Fudan University, Shanghai, 201508, China; b Intelligent Medicine Institute, Fudan University, Shanghai, 200032, China

**Keywords:** COVID-19, Proteomics, Lung, Bioinformatics, Neutrophil extracellular trap formation, Phagosome

## Abstract

The molecular pathology of lung injury in patients with Corona Virus Disease 2019 (COVID-19) remain unclear. In this study, we performed a proteomic study of lung tissues from seven patients with COVID-19, and eight without. Lung parenchymal tissues with COVID-19 were obtained from autopsy samples, while control samples were obtained from paracancerous tissues. Proteins were extracted using phenol extraction. A tandem mass tag-based quantitative proteomic approach combined with bioinformatic analysis was used to detect proteomic changes in the SARS-CoV-2-infected lung tissues. A total of 6,602, and 6,549 proteins were identified in replicates 1 and 2, respectively. Of these, 307, and 278, respectively, were identified as differentially expressed proteins (DEPs). In total, 216 DEPs were identified in this study. These proteins were enriched in 189 Kyoto Encyclopedia of Genes and Genomes (KEGG) pathways. The downregulated proteins are mainly involved in focal adhesion (n = 5), and the PI3K-Akt signaling pathway (n = 4). The upregulated proteins were related to neutrophil extracellular trap (NET) formation (n = 16), and the phagosome pathway (n = 11). The upregulated proteins in these two pathways interact with one another. Further immunohistochemistry verified NET enrichment in the tissues with COVID-19 compared to the controls. Our results systematically outlined the proteomic profiles of the lung's response to SARS-CoV-2 infection and indicated that NET formation was hyper-activated. These results will hopefully provide new evidence for understanding the mechanism behind fatal COVID-19.

## 1 Introduction

Coronavirus disease 2019 (COVID-19), caused by the SARS-CoV-2 virus, has led to ≥ 6.9 million deaths till May 10, 2023 [1,2]. A number of risk factors have been identified to increase COVID-19-associated morbidity including old age, comorbidities (such as cardiomyopathy, diabetes, and chronic respiratory disease), cytokine storm syndrome, and increased number of circulating neutrophils [[3], [4], [5], [6]]. However, the full mechanism underlying COVID-19 mortality still remains to be elucidated. Autopsy-based morphological and molecular studies of pulmonary lesions have somewhat improved our understanding of death caused by COVID-19 [[7], [8], [9]]. The lung, as the main organ targeted by SARS-COV-2, has been widely used in COVID-19 diagnosis, such as through extensive chest computed tomography (CT) scanning. Studies on COVID-19 using lung tissues have also revealed critical genomic [10], proteomic [[11], [12], [13], [14], [15]], and transcriptomic [9,15] features related to the disease. Previous proteomic studies have identified a number of proteins related to COVID-19, involved in immunopathology [12], inflammation and the immune response [13,15,16], and glucose and fatty acid metabolism [11]. However, the molecular changes that take place in infected lung tissues remain elusive. Herein, we report a proteomic profile that we derived by analyzing autopsy lung tissue samples from seven patients who succumbed to COVID-19, as well as eight control tissue samples taken from patients who were COVID-19 free. A total of 6,602, and 6,549 proteins were identified in replicates 1 and 2, respectively. Of these, 307, and 278, respectively, were identified as DEPs. In total, 216 DEPs were identified in this study. Several pathways were altered between the cases and the controls, including ones involved in neutrophil extracellular trap (NET) formation, the lysosome pathway, and the phagosome pathway. The data obtained will hopefully provide fresh insights to aid our understanding of the lung injuries experienced by patients with COVID-19 and suggest some potential new treatment directions.

## 2 Materials and methods

### 2.1 Materials

Tandem Mass Tag (TMT) pro 16-plex, bicinchoninic acid (BCA), protein molecular weight marker, water, and acetonitrile were purchased from ThermoFisher Scientific (Waltham, MA, USA). SDS lysis buffer and phenylmethanesulfonyl fluoride were obtained from Beyotime company (Shanghai, China), while tetraethylammonium bromide, trypsin, and hydroxylamine were sourced from Sigma company (Shanghai, China). Iodoacetamide was supplied by BBI Life Sciences Corporation (Shanghai, China). Dithiothreitol was obtained from Damas-beta company (Shanghai, China), and acetone was from GENERAL-REAGENT (Shanghai, China).

### 2.2 Lung tissue sample collection

Ethical approval for this study was granted by Shanghai Public Health Clinical Center (approval no.: 2023-S077-01). Approval to perform postmortem examinations was granted by either the patients or their legal next of kin. Between May and August of 2022, postmortem examinations were conducted on seven patients with SARS-CoV-2 infection confirmed by polymerase chain reaction (PCR) testing and radiological evidence of pneumonitis. Postmortems were performed at a mean of 10.7 h (standard deviation ± 2.3h) after death. Whole lung tissues were collected following the postmortem procedures. Lung tissue samples were then fixed with formalin, paraffin-embedded, and used for pathological exam. The left tissues were then stored at −80 °C for further use. In this study, left lung tissues from the right upper lobe of the lung were used for proteomics analysis. The eight control samples were derived from lung cancer-adjacent tissues without SARS-CoV-2 infection taken over the same period, from patients who provided written informed consent for the use of their tissues. These tissues were also stored at −80 °C before being used for proteomic analysis.

### 2.3 Pathological examinations of lung tissues

The lung tissue samples were fixed in 10 % formalin, embedded in paraffin, sectioned, and stained with Hematoxylin–Eosin (H-E) for pathological examinations, or Masson staining for collagen and muscle fibers. Masson staining was performed using Masson tricolor dye (BA4079A; Zhuhai Besso Biotechnology Co., Ltd., Guangdong, China) according to the manufacturer's protocol.

Macrophage detection was performed in a BOND-MAX Fully Automated Research Stainer (LeicaBiosystems Company, Wetzlar, Germany) using its CD163 biomarker for immunohistochemical examinations. Briefly, the lung slices were incubated with primary anti-CD163 polyclonal mouse antibody (ZM-0428; Beijing Zhongshan Jinqiao Biotechnology Co., Ltd., Beijing, China), and treated with a BOND Polymer Refine Detection kit (containing a peroxide block, post primary, polymer reagent, diaminobenzidine (DAB) chromogen and Hematoxylin counterstain) (DS9800; LeicaBiosystems) according to the manufacturer's protocol. The stained slices were scanned using an Olympus BX40 light microscope equipped with a logenEPAS9000 (Olympus Corporation, Tokyo, Japan).

### 2.4 Protein extraction, quantification and TMT labeling

The tissues were thoroughly ground in liquid nitrogen. Protein was extracted using a phenol extraction technique as described previously [17]. Protein concentration was determined by BCA (BCA Protein Assay Kit, Thermo Scientific), and verified by SDS-PAGE. Then one-hundreds of protein (every sample) were reduced, alkylated and in-solution digested overnight with trypsin at 37 °C. The digested peptides were reconstituted in 100 mM TEAB and processed using TMTpro reagents (Thermo Fisher, USA). Five microliters of 5 % hydroxylamine were added to stop the reaction for 15 min and then the peptide solution was dried by vacuum. The peptides were labeled with 130 N, 130C, 131 N, 131C, 132 N, 132C, 133 N, and 133C for eight control samples, and 126, 127 N, 127C, 128 N, 128C, 129 N, and 129C tags for seven samples from COVID-19.

### 2.5 Reversed-phase liquid chromatography (RPLC) analysis

In accordance with previously documented methodologies [17,18], the TMT-labeled peptides were subjected to reversed-phase liquid chromatography (RPLC) for separation, and subsequent identification using liquid chromatography-mass spectrometry (LC-MS/MS). Briefly, the procedure involved separating the TMT-labeled peptides using an Agilent 1100 HPLC liquid chromatograph equipped with an Agilent Zorbax Extend-C18 column (2.1 × 150 mm, 5 μm). This separation occurred through a mobile phase system, where mobile phase A consisted of ACN-H_2_O (2:98, V/V), and mobile phase B consisted of ACN-H_2_O (90:10, V/V), flowing at a rate of 300 μL/min. The elution process followed a gradient pattern: from 0 to 8 min, 98 % A; from 8 to 8.01 min, 98 %–95 % A; from 8.01 to 30 min, 95 %–80 % A; from 30 to 43 min, 80 %–65 % A; from 43 to 53 min, 65 %–55 % A; from 53 to 53.01 min, 55 %–10 % A; from 53.01 to 63 min, 10 % A; from 63 to 63.01 min, 10 %–98 % A; and from 63.01 to 68 min, 98 % A. The collection of fractions occurred at 1-min intervals, spanning from 8 to 54 min, resulting in a total of 15 fractions. The eluted fragment was dried by vacuum for mass spectrometry detection.

### 2.6 Liquid chromatography-mass spectrometry (LC-MS/MS) analysis

The dried peptides were resolved, each loaded into the pre-column Acclaim PepMap100 100 μm × 2 cm (RP-C18, Thermo Fisher) and were separated by an analysis column of Acclaim PepMap RSLC,75 μm × 50 cm (RP-C18, Thermo Fisher). This separation took place within an elution system composed of mobile phase A: H_2_O containing 0.1 % formic acid, and mobile phase B: 80 % ACN containing 0.1 % formic acid. The elution condition is 0∼50 min, 2–28 % B; 50–60 min, 28–42 % B; 60–65 min, 42–90 % B; and 65–75 min, 90 % B.

For the purpose of protein identification and quantification, a Q Exactive HF instrument (Thermo Fisher) was employed. Mass spectrometry settings encompassed a full scanning charge/mass ratio range of 350–1500 in the *m*/*z* domain. The collection of MS/MS chromatograms involved the top 20 peaks, utilizing high-energy collision lysis within a data-dependent positive ion mode. Specific MS parameters were configured as follows: collision energy was set to 32 units; MS/MS resolution was maintained at 45,000; automatic gain control was set to 2e^5^; maximum ion injection time was capped at 80 ms; and dynamic exclusion time was set at 30 s. The peptide solutions were loaded to LC-MS/MS for two technical repeats, and the results were named as replicate 1 and replicate 2, respectively.

### 2.7 Protein identification

Protein identification was executed using Proteome Discoverer (v.2.4.1.15) (provided by ThermoFisher Scientific Company), referring to the uniprot-Homo sapiens-9606-2023.2.1.fasta database. The specific search parameters were as follows: for the MS/MS search, a peptide tolerance of 10 ppm and an MS/MS tolerance of 0.02 Da were employed. Full tryptic specificity was enforced, allowing for up to two missed cleavages. Static modifications included TMTpro 16 plex (N-term, K) and carbamidomethyl (C), while dynamic modifications encompassed oxidation (M) and acetyl (N-term). The instrument chosen for analysis was the Q Exactive HF. For protein identification, the stipulated False Discovery Rate (FDR) was maintained at less than 0.01.

### 2.8 Differentially expressed protein identification and functional annotations

The proteins identified in all 15 samples with two replicates were used for further analysis. Proteins displaying differential expression were designated based on the following criteria: a fold change of either ≤0.5 or ≥2.0, and a *P*-value less than 0.05. To visualize the variance between two sample groups (COVID-19 and the controls), we employed principal component analysis (PCA) using R-Studio (version: 4.2.2) (RStudio Inc., MINNESOTA, USA).

The differentially expressed proteins shared across both replicates were subjected to analysis against three databases: Gene Ontology (GO) (Gene Ontology database; http://www.geneonto logy.org/), Kyoto Encyclopedia of Genes and Genomes (KEGG) (Kyoto Encyclopedia of Genes and Genomes database; http://www.genome.jp/kegg/genes.html), and STRING 11.5 for protein–protein interaction analysis (https://string-db.org). The Gene Ontology (GO) entries (biological process [BP], cellular component [CC], molecular function [MF], and Kyoto Encyclopedia of Genes and Genomes (KEGG) pathways were extracted from GO database. A Kappa score of 0.7 was employed as a confidence threshold. Selection of items was based on an FDR (False Discovery Rate) below 0.05. The construction of the protein-protein interaction (PPI) network was executed using Cytoscape v 3.10.1 [19], utilizing the interacting proteins exported from the STRING software as nodes.

Plugin Molecular Complex Detection (MCODE) [20] was utilized to screen submodules from the PPI network constructed by Cytoscape software, with the criteria of degree cutoff = 2, node score cutoff = 0.2, k-core = 2, max depth = 100, and the minimum number of genes 4. The protein nodes were exported from STRING software using the proteins with subcellular component of extracellular exosome (reported from CC analysis). A cytoscape plugin cytoHubba [21] was used to identify the hub proteins by ranked nodes and the maximal clique centrality (MCC) algorithm.

### 2.9 Statistical analysis

For the proteomic study, quantitative data were statistically analyzed using Microsoft Excel 2010 (Microsoft Corp., Washington, USA). DEPs were defined as those with fold changes of ≥2.0 or ≤0.5, at *p* < 0.05. R-Studio (version: 4.2.2) (RStudio Inc., MINNESOTA, USA) was used to analyze the PCA, volcano plot, and clustering data [22]. For immunohistochemical determination, statistical analyses were performed using GraphPad Prism (version 8.3.0) (GraphPad Company, California, USA). The unpaired Student's t-test was used to compare differences between the two groups. Differences were considered statistically significant at *p* < 0.05.

### 2.10 Verification of NETs by immunohistochemical examinations

Immunohistochemical examinations were carried out on both the COVID-19 and the control lung tissues. Each tissue slice was incubated with a primary anti-myeloperoxidase (MPO) polyclonal mouse antibody (GT203207; GENE Company, Shanghai, China) for staining NETs. The slices were then transferred to our BOND RX Fully Automated Research Stainer for further staining and scanning, as described above.

To semi-quantitatively analyze the expression of MPO in lung tissues, each slice was randomly imaged 10 times at 400× magnification. The MPO-positive cells were counted in each image. The ratio of the average number of positive cells in the seven COVID-19 lung tissues (70 images total) to that of in the eight control lung tissues (80 images total) was considered to be the proportional change of MPO expression.

## 3 Results

### 3.1 Clinical characteristics of the patients and pathological examination of lung tissues

This study analyzed 15 total samples, of which seven were autopsy samples from patients who died of COVID-19 and eight were control samples from patients with lung cancer (Table 1). In the COVID-19 group, the patients had a median age of 69.4 ± 22.7 years (range, 26–100 years), Body Mass Index (BMI) of 23.3 ± 2.0, and included five males and two females. In the control group, the patients had a median age of 59.6 ± 11.1 years (range, 36–74 years), BMI of 22.8 ± 1.0, and included four males and four females. Similar comorbidities were present in both groups, including hypertension, diabetes, cerebral infarction, and pathogenic microbial infection. In both groups, nearly 30 % of the patients had received a COVID-19 vaccine, and 50 % reported smoking. Antibiotics had been used in almost all of the patients in both groups. Overall, although the groups had heterogeneous medical backgrounds, their age, sex-distributions, comorbidities, and medications were comparable.

**Table 1:** Overview of the enrolled patient information.

Case ID	Age (years)	Sex	BMI	Disease	Days after first positive PCR (days)^a^	Postmortems (hours)	Smoking	Vaccine	Comorbidities and disease history	Medicines (antibiotic)
C19-1	26	Male	20.8	COVID-19	62	10	None	Yes	Hypertension, diabetes, cerebral infarction	Ceftazidime
C19-2	65	Male	23.9	COVID-19	14	12	Yes	None	HIV infection	Ceftazidime, moxifloxacin, sulperazone
C19-3	72	Male	23.7	COVID-19	44	9	None	None	Hypertension, Alzheimer's disease	Shupushen, Ceftazidime, Meropenem, Teicoplanin
C19-4	64	Male	24.2	COVID-19	32	8	Yes	Yes	Post colon cancer surgery, hysterectomy	Capofenjin, polymyxin, meropenem
C19-5	79	Female	27.1	COVID-19	65	15	None	None	Post right lung malignant tumor; hypertension; post right renal artery stent surgery	Tigecycline, ceftazidime, meropenem
C19-6	80	Male	22.6	COVID-19	85	10	Yes	None	Tuberculosis	Meropenem, Cabozantin, Gualacillin Tazobactam Sodium
C19-7	100	Female	20.7	COVID-19	27	11	None	None	Unilateral nephrectomy	Capofenjin (antifungal), meropenem, tigecycline, polymyxin
Statistical analysis	69.4 ± 22.7	5 males, 2 females	23.3 ± 2.0		47 ± 27.9	10.7 ± 2.3				
Con-1	36	Male	22.5	Cancer	Neg	NA	Yes	Yes	Pneumonia	Cefotiam
Con-2	61	Male	22.8	Cancer	Neg	NA	Yes	None	Diabetes	None
Con-3	63	Male	23	Cancer	Neg	NA	Yes	None	Hypertension, pneumonia	Cefazolin
Con-4	74	Female	22.6	Cancer	Neg	NA	None	None	Hypertension, pneumonia	Cefazolin
Con-5	67	Female	25	Cancer	Neg	NA	None	None	Hypertension, pneumonia	Moxalactam
Con-6	55	Male	22.6	Cancer	Neg	NA	Yes	Yes	HIV infection, pneumonia	Cefoperazone sodium and Subactam sodium for injection
Con-7	58	Female	22.8	Cancer	Neg	NA	None	None	No	None
Con-8	63	Female	21.3	Cancer	Neg	NA	None	None	Hypertension; cerebral infarction, pneumonia	Cefotiam
Statistical analysis	59.6 ± 11.1	4 males, 4 females	22.8 ± 1.0							
P value	0.3		0.6							

a the number of days that patients remained positive after first positive PCR.

**Fig. 1:**
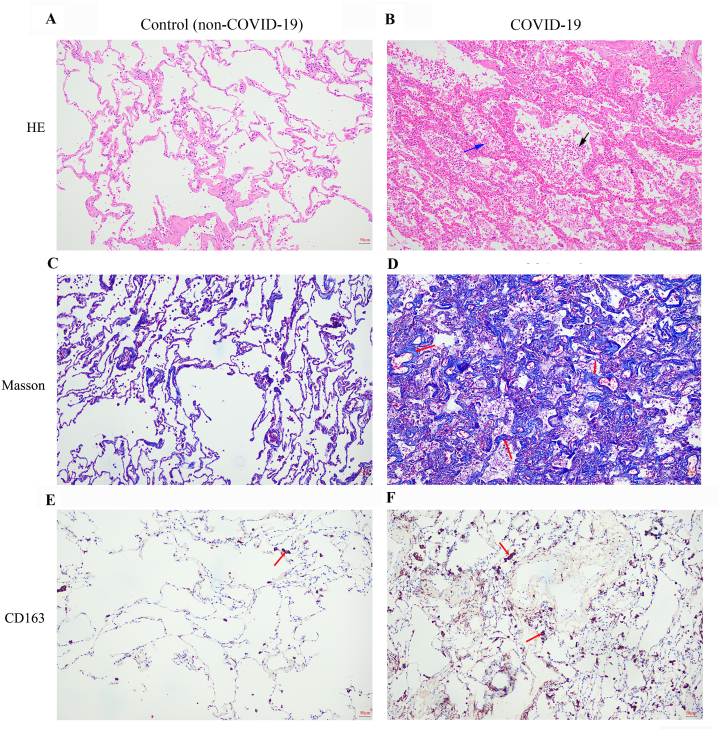
Comparison of histopathological features of lung between COVID-19 and non-COVID-19 patients A and B. the lung tissues stained by hematoxylin–eosin. A. a represent pathological feature of lung from the non-COVID-19 patients. The exfoliated alveolar epithelium and infiltrated macrophages were highlighted by blue arrows, whereas, neutrophil infiltration was highlighted by black arrow. B. a represent pathological feature of lung from the COVID-19 patients. the lung shows that the alveolar epithelium was exfoliated and macrophage exudation (highlighted by green arrow), and neutrophil infiltration to the alveolar cavity (highlighted by black arrow). C. and D. the lung tissues stained by Masson. C, a represent image from the controls, and D. from the COVID-19. The thickened collagen and reticular fibres were highlighted by red arrows. E. and F. the lung tissues immune-stained by CD163. E. from the controls, and F. from the COVID-19. The deeper stained macrophages were highlighted by red arrows. The images were 100 x.

To detect the pathological changes in the patient's tissues, pathological examinations were performed. As shown in Fig. 1B stained by H-E, the COVID-19-affected lungs exhibited exfoliation of the alveolar epithelium, macrophages, and neutrophils into the alveolar cavity. Further Masson staining revealed that collagen and reticular fibers were thickened (indicated by darker blue staining) in the patients with COVID-19 compared to the controls (Fig. 1C and D). Moreover, more and darker-stained macrophages (brownish-yellow of on CD163 staining) were detected in the COVID-19 tissues than in the controls (Fig. 1E and F).

### 3.2 SDS-PAGE

Before starting the proteomic study, an SDS-PAGE experiment was performed to ensure consistent protein loading. Results verified clear protein bands and uniform total protein levels in all 15 samples (Supplemental Fig. 1 (Fig. S1)).

### 3.3 Protein identification

**Fig. 2:**
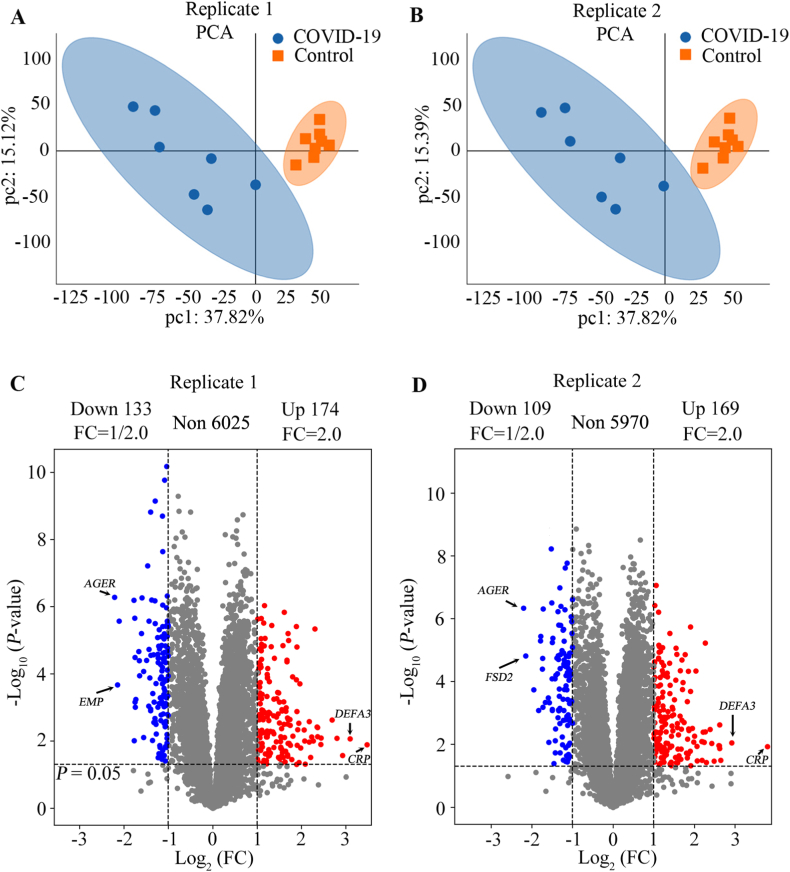
Protein expression analysis by a principal component analysis (PCA) and volcano plot using R-Studio (version: 4.2.2) A and B. PCA of the 15 samples in replicate 1 and 2, respectively; C and D. The differential protein expression analysis visually depicted by a volcano plot. C, for replicate 1; D, for replicate 2. Red, blue and black dots indicated higher, lower and no different expression in the lung tissues from patients with COVID-19 vs. the control. FC, fold change.

The peptides were labeled with TMT kit, and detected twice by LC-MS/MS as shown in Fig. S2. A total of 6,602 (Table S1), and 6,549 proteins (Table S2) were identified in replicates 1 and 2, respectively. Of them, 6,332 and 6,248 were creditable proteins with Score Sequest HT > 0, unique peptide ≥1, and TMT quantification information. PCA revealed that the seven COVID-19 samples and eight control samples were grouped respectively in both replicates 1 and 2. The top two PCA components (PC1: 37.82 % and PC2: 15.12 % in replicate 1; and PC1: 37.88 % and PC2: 15.39 % in replicate 2, respectively, with a 95 % confidence) can distinguish them well (Fig. 2A and 2B).

In this study, differentially expressed proteins were defined as “fold change ≥2 or ≤0.5” and a “*p*-value <0.05” comparing the TMT signal in COVID-19 with that in the controls. A total of 307 (Table S3) and 278 (Table S4) differentially expressed proteins were detected in replicate 1, and replicate 2, respectively. Of them, 133 and 109 proteins, and 174, and 169 proteins were down-, and up-regulated in replicate 1 and 2, respectively (Table S3 and Table S4). According to the volcano plot, these differentially expressed proteins were mostly distributed in 2.0–4.0 fold changes (Fig. 2C and 2D). After analyzed deeper those proteins, we found that C-reactive protein (*CRP*) and Neutrophil defensin 3 (*DEFR*) were the most upregulated proteins in both replicates, with fold changes more than 7.5. While advanced glycosylation end product-specific receptor (*AGER*), and Epithelial membrane protein 2 (*EMP2*) or Fibronectin type III and SPRY domain-containing protein 2 (*FSD2*) were the most down-regulated proteins in replicate 1 and replicate 2, respectively (Table S3, Table S4, and Fig. 2C and 2D). A total of 216 proteins were detected to be common differential proteins, including 88 proteins were down-regulated, and 128 up-regulated (Table S5). As revealed by hierarchical clustering analysis, when judging based on *p*-value <0.05 and fold change (FC) ≤ 0.5, or ≥2.0, the protein expression of the COVID-19 group exhibited significant difference compared with the control group (Fig. S3).

**Fig. 3:**
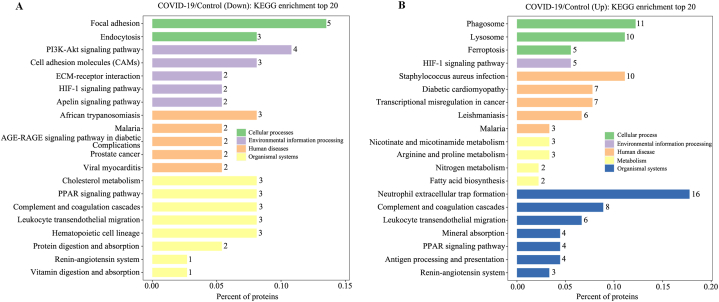
KEGG pathway analysis of the differentially expressed proteins. The top 20 pathways according to KEGG enrichment analysis were shown. A, and B. from downregulated and upregulated proteins, respectively. Green, purple, orange and yellow represent the pathways of cellular processes, environmental information processing, human diseases and organismal systems, respectively. Number of proteins enriched in each pathway was shown in each column.

### 3.4 Functional classification for differentially expressed proteins

**Fig. 4:**
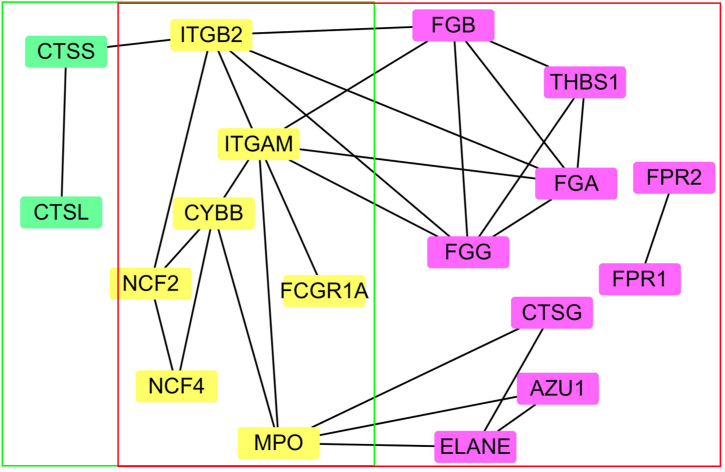
Protein-protein interactions analyzed by STRING software. Proteins in red box were from neutrophil extracellular trap (NET) formation pathway, and green box from phagosome pathway. The proteins only in NET pathway were highlighted in purple background, only in phagosome pathway were highlighted in green background, in the both pathways by yellow background.

The Gene Ontology (GO) entries (biological process [BP], cellular component [CC], and molecular function [MF]) corresponding to the common DEPs were analyzed, and the 10 top entries were sorted in ascending order according to their corresponding −log10 *p*-values. As is shown in Fig. S4, the downregulated proteins were mainly involved in the BP of lipoprotein metabolism including nine out of the top ten items, and nearly 40 % of them were located in the extracellular exosome according to CC analysis. In terms of MF, the downregulated proteins were mainly involved in binding functions (eight out of the top 10). The upregulated proteins were involved in the BPs of proteolysis, inflammatory response, and collagen catabolic process. In terms of CC, the subcellular localizations of about 50 % were to the extracellular exosome. In terms of MF, the DEPs were mainly involved in binding and enzymatic activities. According to our KEGG pathway analysis, these DEPs were enriched in 189 KEGG pathways (Table S6). Of these, the downregulated proteins were mainly enriched in the pathways involving in cellular processes (two items (n = 2)), environmental information processing (n = 5), human diseases (n = 5), and organismal systems (n = 8) (Fig. 3A,Table S7). For the upregulated proteins, they were mainly involved in cellular processes (n = 3), environmental information processing (n = 5), human diseases (n = 4), metabolism (n = 4), and organismal systems (n = 7) (Fig. 3B,Table S8). In particular, 16 upregulated proteins were enriched in the NET formation pathway (Table 2, Fig. 3B, Fig. S5, and Table S8) and 11 were enriched in the phagosome pathway (Table 2, Fig. 3B, Fig. S6, and Table S8). Further protein-protein interaction analysis showed that the 16 DEPs in NET formation pathway had protein-protein interactions with the nine DEPs in phagosome pathway (Fig. 4).

**Table 2:** The up-regulated proteins involved in neutrophil extracellular trap formation and phagosome pathway.

Accession	Gene name	Protein name	*P*value	Fold change
In neutrophil extracellular trap formation pathway				
P02671	FGA	Fibrinogen alpha chain	3.99E-02	3.04
P02675	FGB	Fibrinogen beta chain	2.88E-02	3.12
P02679	FGG	Fibrinogen gamma chain	2.40E-02	3.12
P04839	CYBB	Cytochrome *b*-245 heavy chain	8.12E-05	2.02
P05107	ITGB2	Integrin beta-2	1.26E-03	2.21
P05164	MPO	Myeloperoxidase	3.29E-03	3.54
P08246	ELANE	Neutrophil elastase	3.62E-03	2.56
P08311	CTSG	Cathepsin G	4.48E-04	2.16
P11215	ITGAM	Integrin alpha-M	1.26E-02	2.07
P12314	FCGR1A	High affinity immunoglobulin gamma Fc receptor I	3.48E-03	2.30
P19878	NCF2	Neutrophil cytosol factor 2	6.29E-03	2.03
P20160	AZU1	Azurocidin	3.17E-03	2.16
P21462	FPR1	fMet-Leu-Phe receptor	1.74E-02	2.50
P25090	FPR2	N-formyl peptide receptor 2	8.97E-03	2.66
P30405	PPIF	Peptidyl-prolyl *cis*-trans isomerase F, mitochondrial	1.02E-05	2.25
Q15080	NCF4	Neutrophil cytosol factor 4	7.34E-03	2.16
In phagosome pathway				
P04839	CYBB	Cytochrome *b*-245 heavy chain	7.83E-05	2.03
P05107	ITGB2	Integrin beta-2	1.25E-03	2.19
P05164	MPO	Myeloperoxidase	3.32E-03	3.49
P07711	CTSL	Procathepsin L	7.30E-05	2.23
P07996	THBS1	Thrombospondin-1	3.73E-04	2.82
P11215	ITGAM	Integrin alpha-M	1.29E-02	2.07
P12314	FCGR1A	High affinity immunoglobulin gamma Fc receptor I	3.24E-03	2.33
P19878	NCF2	Neutrophil cytosol factor 2	6.28E-03	2.04
P25774	CTSS	Cathepsin S	2.43E-03	2.85
P35442	THBS2	Thrombospondin-2	1.22E-05	2.71
Q15080	NCF4	Neutrophil cytosol factor 4	6.92E-03	2.17

In this study, in term of subcellular component analysis, we found that proteins enriched in extracellular exosome were the top one for both down-regulated and up-regulated proteins. To discover the proteins driving these opposing trends, a protein-protein interaction analysis was constructed with the proteins with subcellular component of extracellular exosome. Plug-in MCODE analysis discovered that these proteins were enriched in two local network clusters (STRING), including “mixed incl. Neutrophil mediated cytotoxicity, and Mononeuritis multiplex” (named as neutrophil cluster), and “mixed, incl. complement and coagulation cascades, and protein-lipid complex” (named as complement cluster) (Fig. S7A). Then the maximal clique centrality algorithm in the Cytoscape plugin cytoHubba [20,23] was applied to select the top ten hub genes (Fig. S7B). The results indicated that three downregulated proteins (Apolipoprotein C-III (APOC3), Apolipoprotein A-II (APOA2), and Alpha-2-HS-glycoprotein (AHSG)), and seven upregulated proteins (BPI, AZU1, Fibrinogen alpha chain (FGA), Fibrinogen gamma chain (FGG), Cathepsin G (CTSG), Neutrophil elastase (ELANE), and MPO) were involved (Fig. S7C).

### 3.5 NETs were hyper-activated in patients with COVID-19

**Fig. 5:**
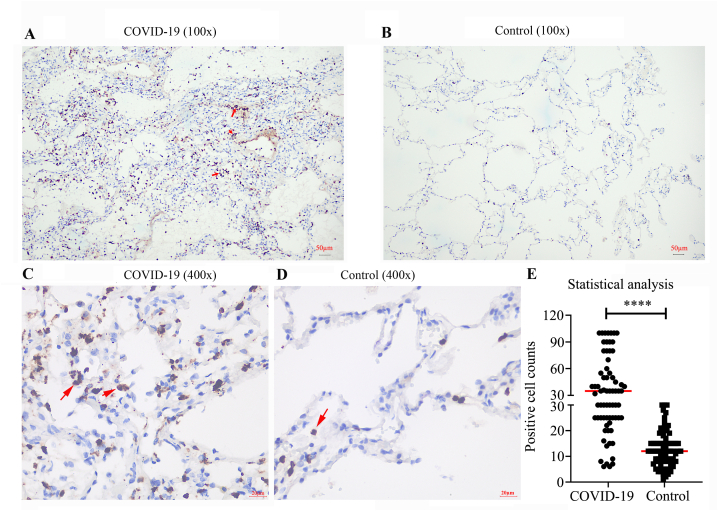
Immunohistochemical examinations and semi-quantification of Myeloperoxidase (MPO) in the patients with COVID-19 and the controls. The lung sections from patients with COVID-19 (COVID-19), or patients with lung cancer (control) were stained with anti-MPO monoclonal antibody. A and B. representative images with 100x from patients with COVID-19 and the controls, respectively. C and D, representative images with 400x from patients with COVID-19 and the controls, respectively. E. semi-quantitative analysis of MPO expression in COVID-19 (n = 7*10) and the control (n = 8*10). Each sample was randomly scanned for 10 images, and used for statistics analysis. Cells were stained for nuclei (DAPI, blue), MPO (brown). The positive cells were highlighted by red arrows.

To confirm our proteomics findings, we performed immunohistochemical examinations using MPO staining. As is shown in Fig. 5, higher levels of NETs (indicated by MPO staining) were found in the lung tissues of the patients with COVID-19 patients (Fig. 5A) compared to the healthy controls (Fig. 5B). Moreover, a semi-quantitative analysis of MPO expression was performed using 400x images (Fig. 5C and D), and showed a significant upregulation in the lung tissues of patients with COVID-19 patients compared to the controls, with positive cell counts of 43.3 ± 28.5 vs 12.6 ± 6.9 (Fig. 5E). These data indicated that NETs were hyper-activated in the lung tissues of patients with severe cases of COVID-19.

## 4 Discussion

A number of proteomic studies have been carried out to discover the molecular and cellular modifications associated with COVID-19 disease [11,[24], [25], [26], [27], [28]]. Most of these were performed using bodily fluids such as plasma [[25], [26], [27],^29^], peripheral blood cells [25,30,31], urine [32], and bronchoalveolar lavage fluid [26]. Few studies on this topic have used lung tissues [11,15,24,33]. As has been shown, lung represent the main target organ for SARS-CoV2, and molecular change in the lung can offer new insights for understanding the mechanism behind COVID-19, as well as potentially provide new drug targets for its treatment. In this study, we performed a proteomic analysis using lung tissues from patients with fatal COVID-19, comparing the findings to the controls from the adjacent normal lung tissues of patients with lung cancer without COVID-19.

A total of 216 DEPs were identified. The downregulated proteins were mainly involved in the BPs of lipoprotein metabolism, while the upregulated proteins were related to proteolysis, the inflammatory response, and the collagen catabolic process. Our findings are similar to those in a previous report, which claimed that COVID-19-associated DEPs in lung tissues were involved in cellular metabolism, blood coagulation, the immune response, angiogenesis, regulation of the cellular microenvironment, and inflammatory processes [13,15,16,33].

According to our KEGG pathway analysis, 16 of the upregulated DEPs in this study were enriched in the NET formation pathway. As is known, elevated neutrophil levels may be caused by viral, and bacterial infection. In our study, all involved patients have secondary bacterial infections in their lungs, and some cases have fungal infections. The primary pathogenic microorganisms identified among these patients comprised Acinetobacter baumannii, *Klebsiella pneumoniae*, *Pseudomonas aeruginosa*, Candida smooth and *Candida tropicalis* (Table S9). An increased number of circulating neutrophils is an indicator of worse COVID-19 outcomes [34]. In patients with COVID-19, NET markers (such as MPO-DNA complexes, and citrullinated histone H3) have been discovered to be overexpressed in the sera, and their levels positively correlate with the severity of COVID-19 [5]. NETs have also been detected at higher levels in the tracheal aspirates and lung autopsies tissues of patients with COVID-19. Furthermore, SARS-CoV-2 can directly induce the release of NETs [6]. However, in previous studies on NETs and COVID-19, NETs or their biomarkers were analyzed. In our study, a high throughout proteomics study was performed and 16 NET-associated proteins were found to be upregulated in COVID-19. Consequently, our study provides a proteomic interpretation of NETs in the context of COVID-19 that complements existing research. Furthermore, according to a previous report, COVID-19 can activate NETs, which then activate IL-1-β, and IL-1-β to form an IL-1β-NET loop [35]. As a result, the neutrophil-lymphocyte ratio has been proposed as a prognostic marker of severity in hospitalized COVID-19 cases [36]. In our study, several NET-related proteins were upregulated. For example, MPO, a protein synthesized in neutrophils and monocytes, was detected to be upregulated for more than 3-fold. Proteins that interact with MPO, such as cytochrome *b*-245 heavy chain (CYBB), neutrophil cytosol factor 2 (NCF2), neutrophil cytosol factor 4 (NCF4), high affinity immunoglobulin gamma Fc receptor I (FCGR1A), integrin alpha-M (ITGAM), and integrin beta-2 (ITGB2), were also discovered to be upregulated. These proteins have been reported to be related to COVID-19. For example, MPO has been found to be upregulated in patients with COVID-19 [11]. NCF4 has been shown to be a risk gene associated with the progression of kidney injury in COVID-19 patients [37]. Soluble ITGAM and ITGB2 elevation have also been shown to correlate significantly with long-term pulmonary COVID-19 complications and may represent promising biomarkers for predicting such complications [38]. CYBB was found to be elevated in the peripheral blood mononuclear cells of patients with COVID-19 [39]. FCGRIA has been found to be a hub protein in COVID-19 [40], and has been proposed as an attractive target for immunotherapy [41]. Therefore, our study expanded on previous observations concerning the involvement of NETs in COVID-19 [5,6,11,35,36].

In this study, the second and third most activated pathways were found to be the phagosome and lysosome ones, respectively. These included 11 upregulated proteins (cytochrome *b*-245 heavy chain (CYBB), integrin beta-2 (ITGB2), MPO, procathepsin L (CTSL), thrombospondin-1 (THBS1), ITGAM, FCGR1A, NCF2, cathepsin S (CTSS), thrombospondin-2 (THBS2), and NCF4) from the phagosomal pathway, and 10 upregulated proteins (lysosomal acid glucosylceramidase (GBA1), cathepsin D (CTSD), CTSL, cathepsin B (CTSB), CTSG, lysosomal protective protein (CTSA), cathepsin E (CTSE), CTSS, legumain (LGMN), and cathepsin Z (CTSZ)) from the lysosomal one. Pathogenic microorganisms can stimulate the host phagosome to take up microbes [42]. Phagosome‒lysosome fusion is an important process that is involved in killing of the intracellular pathogens [43]. A previous study also found that the proteins overexpressed in COVID-19 (compared to healthy controls) were enriched in the lysosomal and phagosomal pathways [44]. Only CTSS, however, was detected in both that study [44] and this one. This difference may have been due to the different samples (airway mucus from adults with severe COVID-19 vs lung tissues from fatal patients with COVID-19) and differential proteomic analysis methods (label-free vs. TMT labeling).

We also found that the complement and coagulation cascade pathway was activated, with eight proteins being upregulated (FGA, fibrinogen beta chain (FGB), FGG, ITGB2, ITGAM, integrin alpha-X (ITGAX), urokinase plasminogen activator surface receptor (PLAUR), and V-set and immunoglobulin domain-containing protein 4 (VSIG4)). These upregulated proteins were enriched in the complement and coagulation pathways, which was consistent with hyper-activation of the immune response. Similarly, in previous reports, FGB, FGG, complements C4, C3, C5, C2, C9 [13], and complement C1q subcomponent subunits A, B, and C (C1QA, C1QB and C1QC) [16] were found to be upregulated in the lung tissues of patient with COVID-19. Not all of the DEPs identified in this study had been detected in previous studies [13,16]. Therefore, in terms of complement and coagulation cascade pathways, our study can contribute some new dysregulated proteins in patients with COVID-19.

In this study, in term of subcellular component analysis, we found that proteins enriched in extracellular exosome were the top one for both down-regulated and up-regulated proteins. To discover the proteins driving these opposing trends, subnetwork and the top ten hub proteins were extracted. Our results indicated that AHSG (downregulated), FGA (upregulated), and FGG (upregulated) might drive these opposing trends, because they were the top three proteins ranked by MCC method, and key nodes bridging the upregulated and downregulated proteins (seen from Fig. S7). According to the annotation from the database of uniprot (https://www.uniprot.org/uniprotkb), AHSG can promotes endocytosis. FGA together with FGB, and FGG can polymerize to form an insoluble fibrin matrix. Fibrin deposition is associated with infection, where it protects against IFNG-mediated hemorrhage. The reports about AHSG, FGA and FGG were very limited [45,46]. The deep mechanism of these three proteins regulating SARS-CoV-2 infection need to be studied in the future.

This study was subjected to several key limitations worth noting. First, the sample size was small, with only seven COVID-19 samples, and eight non-COVID-19 controls. Owing to the relatively small number of patients, no stratified research (such as comorbidities, age, and drugs) was performed. The results of this study therefore warrant confirmation through larger independent cohort studies. Second, the control samples were only histologically healthy because they were paracancerous tissues surgically resected from patients, as has been done in related studies [11]. Third, we did not enroll patients died of pneumonia but did not have COVID-19, therefore, the results from this study may not be completely COVID-specificity. Fourth, in this study, patients with COVID-19 have secondary bacterial infections in their lungs, therefore, it is unclear if the results are COVID-19 specific or only due to the alveolar bacterial infection. In the future, it is important to perform experiments to verify the expression of DEPs in the patients with or without the alveolar bacterial infections. Fifth, future verification and in-depth investigation of DEPs that we identified particularly the upregulated NET pathway proteins are warranted.

In summary, we identified 216 DEPs in lung tissues from patients who died from COVID-19, compared to control patients without COVID-19. This proteomic atlas uncovered multiple biological and pathological processes that may be dysregulated during COVID-19, which include but not limited to lipoprotein metabolism, proteolysis, the inflammatory response, and the collagen catabolic process. In particular, we found that the NET formation pathway was highly enriched. The results of this study will hopefully provide new insights into how the lung proteome is involved in COVID-19, and further our understanding of COVID-19 pneumonia.

## Ethics and consent statement

All protocols for this study were reviewed and approved by Shanghai Public Health Clinical Center (2023-S077-01, dated on July 4, 2023). Consent approval to perform postmortem examinations was granted by either the patients or their legal next of kin.

## Funding

This study was supported by Science and Technology Plan Project of Zhejiang (2022C03189), and three Year Action Plan for Promoting Clinical Skills and Clinical Innovation in Municipal Hospitals from 10.13039/501100014137Shanghai Shenkang Hospital Development Center (SHDC2022CRS024B).

## Data availability statement

The data to support the results are available in the manuscript as supplement results.

The datasets presented in this study can be found in online repositories. The names of the repositories and accession numbers are available at http://www. iprox.cn/, PXD045427. F1, 2, 3, 4, 6, 7, 8, and 9 in PXD045427 were from the control group, and named as Control-1, 2, 3, 4, 5, 6, 7 and 8 in this manuscript, respectively. C19–1, C19–2, C19–3, C19–4, C19–5, C19-6, and C19-7 represent the sample 1 to 7 from the patients with COVID-19.

## CRediT authorship contribution statement

**Shu Song:** Writing – original draft, Resources, Methodology, Data curation. **Liyan Zeng:** Writing – original draft, Software, Methodology, Data curation. **Jingjing Xu:** Resources, Methodology. **Lei Shi:** Visualization, Resources. **Lingqing Lu:** Methodology. **Yun Ling:** Writing – review & editing, Project administration, Funding acquisition, Conceptualization. **Lijun Zhang:** Writing – review & editing, Writing – original draft, Validation, Supervision, Resources, Project administration, Methodology, Investigation, Funding acquisition, Formal analysis, Data curation, Conceptualization.

## Declaration of competing interest

The authors declare that they have no known competing financial interests or personal relationships that could have appeared to influence the work reported in this paper.

## Figures and Tables

**fig s1:**
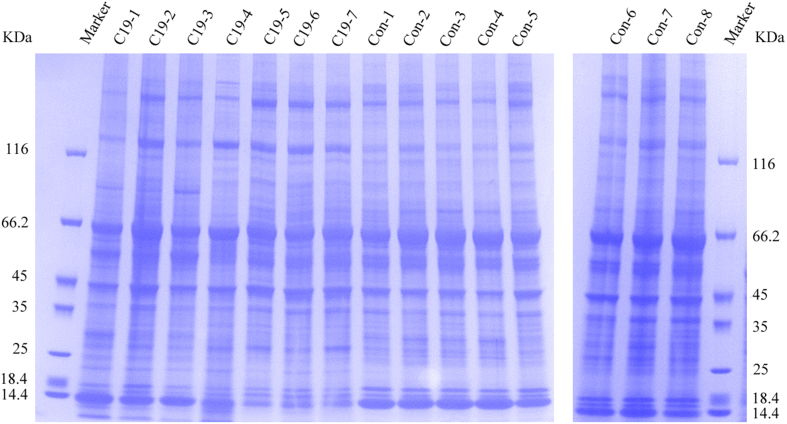


**fig s2:**
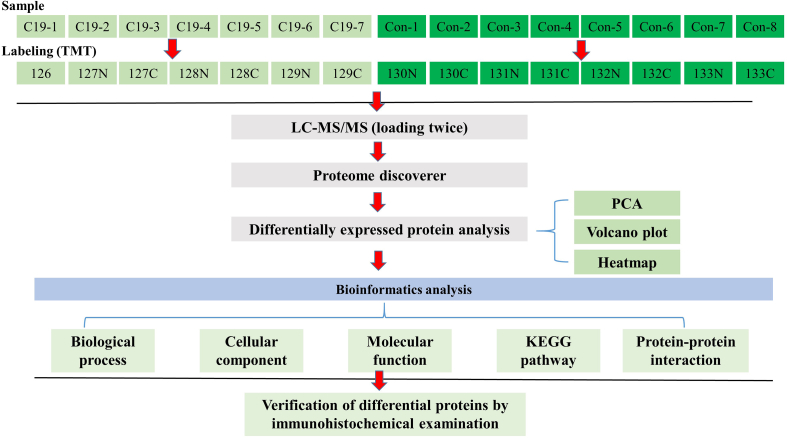


**fig s3:**
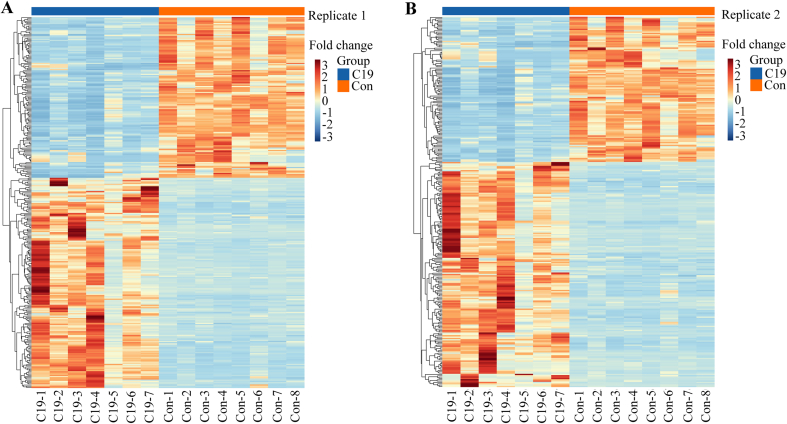


**fig s4:**
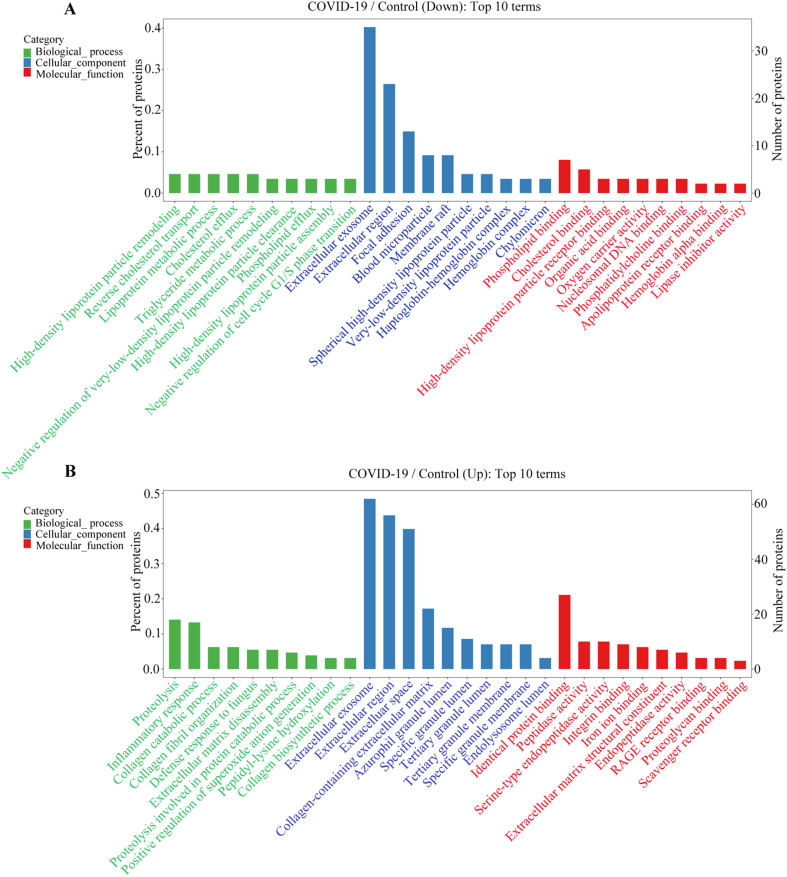


**fig s5:**
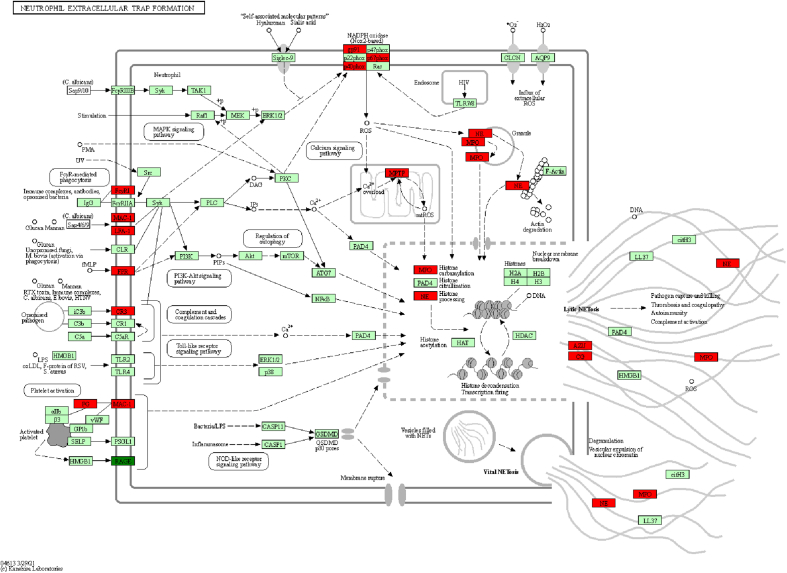


**fig s6:**
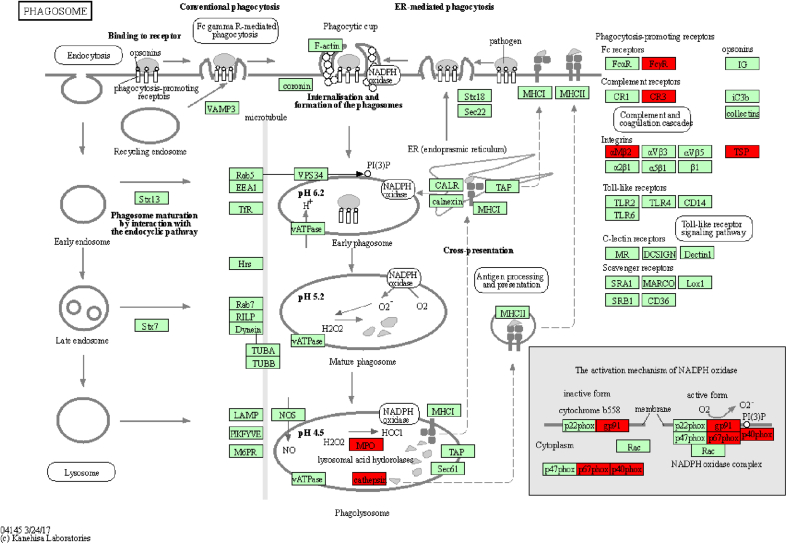


**fig s7:**