# Supplementary material for: Proteomic lung analysis revealed hyper-activation of neutrophil extracellular trap formation in cases of fatal COVID-19
Source: Heliyon. 2024 May 24;10(11):e31878. doi: 10.1016/j.heliyon.2024.e31878 (PMC11177151; doi:10.1016/j.heliyon.2024.e31878)
Supplement: Multimedia component 9 [file mmc9.docx]

**Table S9** The clinical characteristic of the seven COVID-19 patients

| Number | Comorbidities and disease history | Pulmonary pathology | Pathogenic microorganism |
| --- | --- | --- | --- |
| 1 | Hypertension, diabetes, cerebral infarction | Pulmonary infection | *SARS-CoV2* |
| 2 | HIV-infection | Pulmonary infection, sepsis, and septic shock. | *SARS-CoV2*, *Pseudomonas aeruginosa, Klebsiella pneumoniae, and Gram positive cocci* |
| 3 | Hypertension, Alzheimer's disease | Pulmonary infection, sepsis. | SARS-CoV2, *Klebsiella pneumoniae, Escherichia coli, Gram positive cocci, Gram negative cocci, Stenotrophomonas maltophilia, Neisseria meningitidis, fungal spores, Candida smooth* |
| 4 | Post colon cancer surgery, hysterectomy | Pulmonary infection. | *SARS-CoV2*, *Candida smooth, Klebsiella pneumoniae, Enterococcus faecalis, Enterobacter aeruginosa* |
| 5 | Post right lung malignant tumor, hypertension, post right renal artery stent surgery | Interstitial lung disease, pulmonary infection. | *SARS-CoV2*, *Acinetobacter baumannii, Gram positive cocci* and *Gram negative cocci* |
| 6 | Tuberculosis | Pulmonary tuberculosis, bilateral pulmonary infection, septic shock. | *SARS-CoV2*, Mycobacterium tuberculosis, Acinetobacter baumannii, Klebsiella pneumoniae, Candida tropicalis, Gram positive cocci |
| 7 | Unilateral nephrectomy | Pulmonary infection, sepsis. | *SARS-CoV2*, Candida smoothii, Stenotrophomonas maltophilia, Acinetobacter baumannii, fungal spores, Candida tropicalis |
